# Smartphone Ownership and Usage Among Pregnant Women Living With HIV in South Africa: Secondary Analysis of CareConekta Trial Data

**DOI:** 10.2196/43855

**Published:** 2023-06-22

**Authors:** Sandisiwe Noholoza, Tamsin K Phillips, Sindiswa Madwayi, Megan Mrubata, Carol S Camlin, Landon Myer, Kate Clouse

**Affiliations:** 1 Division of Epidemiology and Biostatistics School of Public Health University of Cape Town Cape Town South Africa; 2 Department of Obstetrics Gynaecology & Reproductive Sciences University of California San Francisco, CA United States; 3 Vanderbilt University School of Nursing Vanderbilt University Nashville, TN United States; 4 Vanderbilt Institute for Global Health Nashville, TN United States

**Keywords:** HIV, mHealth, mobile phone, ownership, smartphone, South Africa

## Abstract

**Background:**

Mobile health (mHealth) initiatives are increasingly common in low-resource settings, but the appropriateness of smartphone interventions in health care settings is uncertain. More research is needed to establish the appropriateness and feasibility of integrating new mHealth modalities (novel apps and social media apps) in the South African context.

**Objective:**

In this study, to inform future mHealth interventions, we describe smartphone ownership, preferences, and usage patterns among pregnant women living with HIV in Gugulethu, South Africa.

**Methods:**

We screened pregnant women living with HIV from December 2019 to February 2021 for the CareConekta trial. To be enrolled in the trial, respondents were required to be 18 years of age or older, living with HIV, ≥28 weeks pregnant, and own a smartphone that met the technical requirements of the CareConekta app. In this secondary analysis, we describe mobile phone ownership and sociodemographic characteristics of all women screened for eligibility (n=639), and smartphone use patterns among those enrolled in the trial (n=193).

**Results:**

Overall, median age was 31 (IQR 27-35) years. Of the 582 women who owned smartphones, 580 responded to the question about whether or not it was a smartphone, 2 did not. Among those with smartphones, 92% (421/458) of them used the Android operating system of version 5.0 or above, 98% (497/506) of phones had a GPS, and 96% (485/506) of individuals charged their phones less than twice a day. Among women who were enrolled in the trial, nearly all (99%, 190/193) owned the smartphone themselves; however, 14% (26/193) shared their smartphone with someone. In this case, 96% (25/26) reported possessing the phone most of the day. Median duration of ownership of the smartphone was 12 (IQR 5-24) months, median duration with current phone number use was 25 (IQR 12-60) months, and median number of cell phone numbers owned 2 years prior to enrollment in the trial was 2 (IQR 1-2). Receiving (192/193, 99.5%) and making (190/193, 99%) phone calls were among the most common smartphone uses. The least used features were GPS (106/193, 55%) and email (91/193, 47%). WhatsApp was most frequently reported as a favorite app (181/193, 94%).

**Conclusions:**

Smartphone ownership is very common among pregnant women living with HIV in this low-resource, periurban setting. Phone sharing was uncommon, nearly all used the Android system, and phones retained sufficient battery life. These results are encouraging to the development of mHealth interventions. Existing messaging platforms—particularly WhatsApp—are exceedingly popular and could be leveraged for interventions. Findings of moderate smartphone ownership turnover and phone number turnover are considerations for mHealth interventions in similar settings.

**Trial Registration:**

ClinicalTrials.gov NCT03836625; https://clinicaltrials.gov/ct2/show/NCT03836625?term=NCT03836625

## Introduction

### Background

Mobile health (mHealth) interventions for the improvement of public health are increasing in popularity globally. The proliferation of mobile phone ownership in the past 2 decades, especially in lower-income countries, has been a massive catalyst for mHealth, with an estimated 7.26 billion of the world’s population owning mobile phones in 2022 [1-3]. mHealth interventions range from simple SMS text message interventions to multi-interface mobile apps that can be used to disseminate health information, facilitate treatment adherence, improve patient engagement and retention, and collect patient data [4-8].

### HIV and mHealth Interventions

In 2021, a total of 7.7 million people in South Africa were living with HIV, the largest HIV epidemic in the world [9]. South Africa is making progress toward meeting the United Nations 95-95-95 targets but gaps in the HIV care cascade persist. In 2021, overall 94% of people living with HIV in South Africa knew their status, 74% of those were on treatment; and 67% of those on treatment were also virally suppressed [10]. Recent research has shown that pregnant and postpartum women living with HIV are at increased risk of dropping out of HIV care [11,12]. mHealth interventions seek to address these gaps through information, adherence support, stigma mitigation, and engagement in HIV care through sustained communication with the health facility [2,13-15].

SMS text messaging is widely available at a low cost to the recipient and is accessible to those who use even the most basic mobile phones [16-21]. SMS text messaging interventions have been used to improve engagement in HIV care and adherence in lower-income countries, including South Africa, through clinic appointments and medication reminders, health education messages, and case management [16-21]. Smartphone interventions, including the use of social media apps, expand the reach of mHealth beyond simple texting, particularly to young people who remain at high risk of HIV infection and poor HIV treatment outcomes [22,23].

### mHealth Barriers and Gaps

Kruse et al [24] noted that barriers to mHealth implementation fall under three categories: lack of infrastructure, lack of equipment, and the technology gap. To make informed evidence-based decisions in the design and implementation of mHealth interventions, it is crucial to understand mobile phone ownership and use patterns in the intended target population within the context of these barriers. The rapid acceleration of technology impacts the access, feasibility, efficacy, and sustainability of current and future mHealth interventions. Therefore, the most current data on mobile phone or smartphone ownership patterns is necessary to improve current mHealth interventions and to inform future mHealth interventions that target various steps in the HIV cascade.

### Research Aim

This research aims to describe smartphone ownership, preferences, and usage patterns among pregnant women living with HIV in South Africa in order to inform future interventions. Given the known successes of simpler mHealth interventions, more detailed and current research is needed to establish the appropriateness and feasibility of integrating new mHealth modalities (novel apps and social media apps) in the South African context. Mitigating this gap in knowledge within the context of each target population has the potential to inspire the design of more targeted mHealth interventions, with potential for greater reach, uptake, and long-term success.

## Methods

### Study Design and Setting

This is a secondary analysis of data collected during the CareConekta randomized controlled trial (ClinicalTrials.gov: NCT03836625). The trial assessed the feasibility, acceptability, and initial efficacy of using CareConekta, a smartphone app designed to facilitate engagement in HIV care by enabling users to locate nearby antiretroviral therapy facilities in times of mobility. The parent trial recruited pregnant women living with HIV from the Gugulethu Midwife Obstetric Unit (MOU), a public antenatal care (ANC) clinic based in the community of Gugulethu in Cape Town, South Africa. This is a high HIV burden setting, with an estimated antenatal HIV prevalence of 30% [25]. Participants were enrolled in the third trimester of pregnancy and followed through 6 months post partum. In this analysis, using data from the screening and enrollment visit, we describe smartphone ownership patterns and use among screened eligible and ineligible pregnant women living with HIV.

### Study Recruitment and Eligibility

All pregnant women living with HIV attending the Gugulethu MOU interested in learning about the CareConekta study were screened for eligibility. Eligibility criteria included being at least 18 years of age, pregnant (≥28 weeks gestation), living with HIV, able to speak and understand isiXhosa (predominant local language) and English, currently owning a smartphone that met certain technical requirements, willing to opt-in to app installation and mobility tracking, and able to demonstrate basic smartphone literacy. For the purposes of the parent trial, a smartphone was defined as a mobile phone with a touchscreen interface and internet and GPS capabilities. In order to enroll, eligible women needed to have their phone available with them at the time of enrollment. Specific technical requirements for enrollment that were assessed at screening included whether the phone had the Android operating system version 5.0 or later, connection to one of the 4 major cell phone service providers (Vodacom, Cell-C, Telkom, or MTN), able to demonstrate use of GPS by opening a map app and finding the current location, and requiring charging less than twice a day (by self-report).

### Ethics Approval

All study activities were approved by the ethical review boards at Vanderbilt University (181640), University of Cape Town (659/2018), and the University of California, San Francisco (237757). Respondents screened for study eligibility gave verbal consent for their data to be recorded in the eligibility checklist forms and to capture basic demographic information if they were ineligible. No identifying information was collected during eligibility screening. Participants who were subsequently enrolled in the trial provided written informed consent. Among enrolled participants, identifying information was maintained in locked storage cabinets at the study site and all electronic documents were password-protected. Participants were assigned study identification numbers to maintain anonymity across study questionnaires. To observe participant privacy and confidentiality protection, this analysis made use of deidentified data from the trial. No compensation was offered to participants for eligibility screening. Individuals enrolled in the study received ZAR 150 (approximately US $8.50) per study visit (enrollment and follow-up) for transport and missed opportunity costs, following standards set by the local human research ethics committee.

### Measures

In this paper, we report a secondary data analysis using the eligibility screening data of all women who were screened for CareConekta, as well as enrollment visit data from all those who were enrolled in the trial. When we launched the parent trial, there were some problems with the completion of the eligibility screening form. When this was realized, we retrained our staff and excluded the forms for this period (n=22) from this analysis.

An eligibility checklist form was completed to determine eligibility (Multimedia Appendix 1). On this form, those found ineligible, or who were eligible but declined participation, were asked to answer a short list of demographic questions following verbal consent. The eligibility checklist was designed to administer all questions on the form, even after the earliest indication of being ineligible. However, administration of this was not always consistent; thus, 18% (115/639) of participants included in this analysis did not complete all the screening questions. Consequently, there is variation in sample size between each variable on the eligibility checklist.

Eligible women willing to participate in the trial completed written informed consent and were enrolled in the CareConekta study. Enrollment was followed by an interviewer-administered questionnaire (see Multimedia Appendix B in Multimedia Appendix 1), which collected similar demographic information and asked about smartphone ownership and usage. All study measures were available in English and isiXhosa.

### Data Analysis

First, to describe the mHealth landscape among the full population of pregnant women living with HIV attending care at the MOU, we compared the smartphone ownership characteristics of those who were enrolled in the CareConekta trial (ie, met all the smartphone-related eligibility criteria) and those who were not enrolled. Those who were not enrolled included all those who did not meet the CareConekta eligibility criteria and those who were eligible but did not enroll on the day of screening, either because they were not interested, were referred to another facility, or were not available to enroll on the day of screening.

Next, to explore differences in sociodemographic characteristics among those enrolled versus those not enrolled, we present data grouped by enrollment status, and further by smartphone ownership status: those who owned smartphones versus those who did not (including those who did not own a mobile phone at all).

Last, to explore smartphone usage and preferences among women enrolled in the CareConekta trial, we present questionnaire responses related to phone ownership and sharing patterns, common phone uses and popular apps, cell phone and cell number turnover, and common reasons for the phone being “off” for extended periods of time.

Data were analyzed using STATA (version 14.2; StataCorp). Descriptive statistics were used to describe the baseline characteristics of all screened women as well as smartphone ownership and usage patterns, presented as frequencies and proportions for categorical variables and as medians, ranges, and IQRs for continuous variables.

Bivariate analyses were conducted using the Wilcoxon rank-sum test for the numerical variables and the chi-square or Fisher exact tests for categorical variables. For all analyses, a threshold *P* value of .05 and 95% CIs were used to determine statistical significance.

## Results

**Figure 1:**
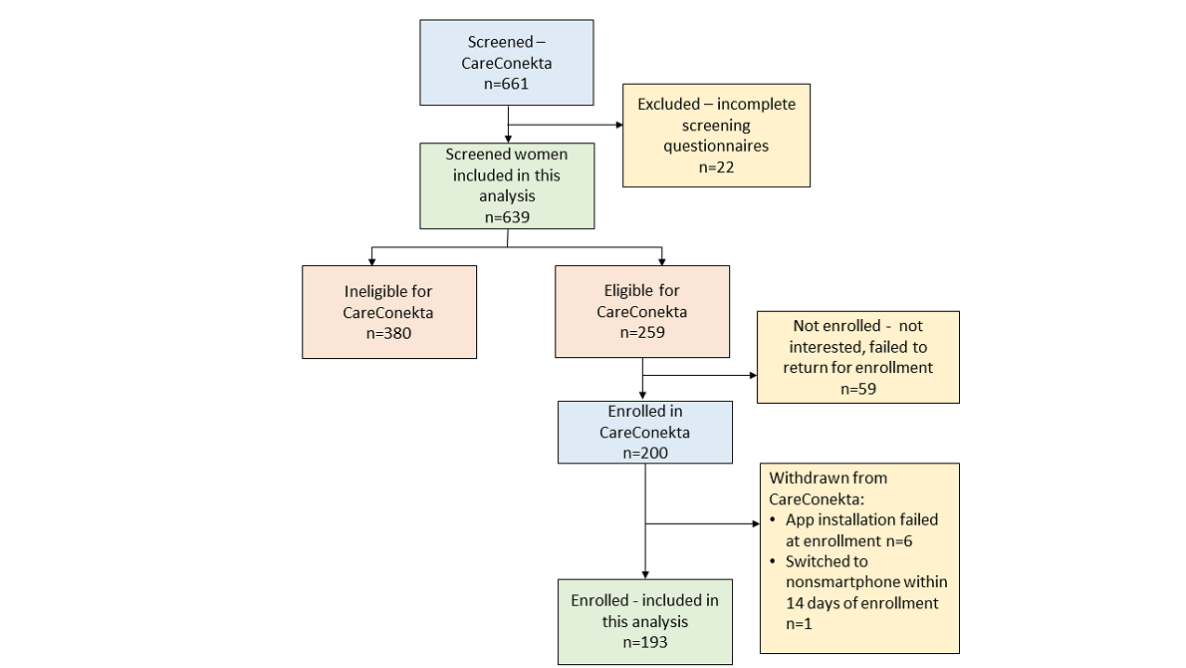
A flowchart illustrating screening and enrollment in the CareConekta trial and illustrating the respondents that are included in this secondary analysis.

As illustrated in Figure 1, a total of 661 pregnant women living with HIV were screened for enrollment into the trial between December 2019 and February 2021. As mentioned, we excluded the screening data of the first 22 women to be screened due to incomplete data. Therefore, a total of 639 women were included in this analysis. Overall, 200 women were enrolled in the CareConekta trial. However, women for whom app installation failed at enrollment (n=6) and those who no longer met the eligibility criteria within 14 days of enrollment (n=1) were withdrawn from the trial and are excluded from the analysis of enrolled participants (n=193).

### Smartphone Ownership Patterns and Determinants of Eligibility for the CareConekta Trial

Table 1 details the determinants of eligibility for the CareConekta trial. Of 639 women included in this analysis, 259 (40.5%) met eligibility criteria and 380 did not. Among those eligible for the trial, 59 were not enrolled (because they were either not interested, needed time to think about enrolling, or promised to return on another day for enrollment).

Among screened women (n=639), age ranged from 16 to 45 years, with a median age of 31 (IQR 27-35) years. Most women screened owned mobile phones (582/637, 91.4%) and 86.7% (503/580) of those owned smartphones. Among those who owned smartphones and responded to the relevant “smartphone feature” questions, 91.9% (421/458) of them owned phones with an Android operating system version 5.0 or above, all used one of the 4 most common local network service providers, 98.2% (497/506) of them used phones that had a GPS feature, 95.9% (485/506) of them charged their phones less than twice a day, and 86.4% (438/507) of them had their phones with them at the time of screening.

Among those ineligible for the trial (n=380), being outside the gestational age window for the trial was the most common reason for ineligibility (226/380, 59.6%), followed by not having the smartphone with them at the time of screening (69/380, 27.8%), and the phone not being a smartphone (77/380, 24.0%).

**Table 1:** Determinants of eligibility and smartphone ownership patterns among 639 women who were screened for the CareConekta study.

Variable			Total participants n=639		Ineligible participants n=380
**Third trimester (n=638)**, n (%)					
	Yes	412 (64.6)		153 (40.4)	
	No	226 (35.4)		226 (59.6)	
**English/IsiXhosa understanding (n=637)**, n (%)					
	Yes	619 (97.2)		360 (95.2)	
	No	18 (2.8)		18 (4.8)	
Age (years), median (IQR)			31 (27-35)		31 (26-35)
**Mobile phone ownership (n=637)**, n (%)					
	Yes	582 (91.4)		323 (85.4)	
	No	55 (8.6)		55 (14.6)	
**Smartphone ownership (n=580)**, n (%)					
	Yes	503 (86.7)		244 (76.0)	
	No	77 (13.3)		77 (24.0)	
**Android operating system version 5.0 or later (n= 458)**, n (%)					
	Yes	421 (91.9)		162 (81.4)	
	No	37 (8.1)		37 (18.6)	
**Service provider (Vodacom, Cell-C, Telkom, or MTN**; **n=506)**, n (%)					
	Yes	504 (99.6)		245 (99.2)	
	No	2 (0.4)		2 (0.8)	
**GPS-enabled phone (n=506)**, n (%)					
	Yes	497 (98.2)		238 (96.4)	
	No	9 (1.8)		9 (3.6)	
**Battery strength (n=506)**, n (%)					
	Charged < twice a day	485 (95.9)		226 (91.5)	
	Charged > twice a day	21 (4.1)		21 (8.5)	
**Phone with participant at screening (n=507)**, n (%)					
	Yes	438 (86.4)		179 (72.2)	
	No	69 (13.6)		69 (27.8)	

### Sociodemographic Characteristics of Women Who Own Versus Those Who Do Not Own Smartphones

We had complete sociodemographic data available for 550 screened women, including 193 women enrolled in the trial. Table 2 shows women’s sociodemographic characteristics, stratified by smartphone ownership status. Overall, 76.5% (421/550) of women owned a smartphone. Median age was 31 years in both groups (*P*=.24). Unemployment was high overall, but significantly higher among those who did not own a smartphone (95/129, 73.6%) compared to those who did (262/421, 62.4%; *P*=.02). Most women had completed some high school education; those who owned smartphones were more likely to have completed high school (35.2%, 147/421 vs 29.5%, 38/129; *P*=.03). Two-thirds of participants (353/550) were born outside of the Western Cape province (where the study clinic was located), with no difference by smartphone ownership (*P*=.96).

**Table 2:** Sociodemographic characteristics among women who were screened for the CareConekta study, by enrollment status and smartphone ownership.

Variable		No smartphone (n=129)	Owns smartphone (n=421)	*P* value	
Age in years, median (IQR)		31 (26-35)	31 (27-35)	.24	
**Employment, n (%)**					.02
	Employed	34 (26.4)	158 (37.6)		
	Unemployed	95 (73.6)	262 (62.4)		
**Education, n (%)**					.03
	Never completed primary school	4 (3.1)	2 (0.4)		
	Completed primary school only	7 (5.4)	25 (6)		
	Some high school	78 (60.5)	222 (53.1)		
	Completed high school	38 (29.5)	147 (35.2)		
	Any tertiary education	2 (1.5)	22 (5.3)		
**Birthplace, n (%)**					.96
	In Western Cape	47 (36.4)	148 (35.3)		
	In South Africa, but not Western Cape	79 (61.2)	261 (62.3)		
	Outside of South Africa	3 (2.3)	10 (2.4)		

### Smartphone Ownership and Use Patterns Among Women Enrolled in the CareConekta Study

Table 3 describes smartphone ownership and use patterns among the 193 enrolled participants. Most participants (190/193, 98.5%) owned their own smartphones. A minority of participants (26/193, 13.5%) shared their smartphone with someone; of these, phone sharing was most common with a romantic partner (20/26, 71.4%), followed by a family member (8/26, 28.6%). In these cases, nearly all participants (25/26, 96.2%) reported having the phone with them most of the day. Median duration of ownership of the particular smartphone used at enrollment was 12 (IQR 5-24) months, median duration of use of the phone number at enrollment was 25 (IQR 12-60) months, and median number of cell phone numbers participants owned in the 2 years prior to enrollment was 2 (IQR 1-2).

Among screened women (n=639), age ranged from 16 to 45 years, with a median age of 31 (IQR 27-35) years. Most women screened owned mobile phones (582/637, 91.4%) and 86.7% (503/580) of those owned smartphones. Among those who owned smartphones and responded to the relevant “smartphone feature” questions, 91.9% (421/458) of them owned phones with an Android operating system version 5.0 or above, all used one of the 4 most common local network service providers, 98.2% (497/506) of them used phones that had a GPS feature, 95.9% (485/506) of them charged their phones less than twice a day, and 86.4% (438/507) of them had their phones with them at the time of screening.

Among those ineligible for the trial (n=380), being outside the gestational age window for the trial was the most common reason for ineligibility (226/380, 59.6%), followed by not having the smartphone with them at the time of screening (69/380, 27.8%), and the phone not being a smartphone (77/380, 24.0%).

**Table 3:** Smartphone use patterns among women who were enrolled in the CareConekta study (n=193).

Variable		Value
**Phone ownership, n (%)**		
	Owned by participant	190 (98.5)
	Owned by intimate partner	2 (0.5)
	Owned by family member	1 (1.0)
**Phone sharing, n (%)**		
	Phone not shared	167 (86.5)
	Phone shared	26 (13.5)
**Person phone is shared with (n=26)^a^, n (%)**		
	Romantic partner	20 (71.4)
	Family member	8 (28.6)
**Person who has phone most of the day if shared (n=26), n (%)**		
	Participant	25 (96.2)
	Someone else	1 (3.8)
Duration of phone ownership (months), median (IQR)		12 (5-24)
Duration of cell number use (months), median (IQR)		25 (12-60)
Number of cell phone numbers in past 2 years, median (IQR)		2 (1-2)
**Phone use, n (%)**		
	Receive phone calls	192 (99.5)
	Receive SMS text messages	191 (99.0)
	Send SMS text messages	191 (99.0)
	Make phone calls	190 (98.5)
	Send “please call me”	184 (95.3)
	Instant messaging (ie, WhatsApp and Facebook messenger)^a^	182 (94.3)
	Photos	180 (93.3)
	Internet	146 (75.7)
	Email	91 (47.2)
	GPS (maps)	106 (54.9)
	Other	74 (38.3)
**Favorite apps^a^, n (%)**		
	WhatsApp	181 (93.8)
	Facebook	112 (58.0)
	Instagram	10 (5.2)
	Twitter	5 (2.6)
	Other	35 (18.1)

^a^More than 1 response was allowed; thus, numbers sum to over 100%.

## Discussion

As mHealth studies continue to grow worldwide, including in low-resource settings, it is important to examine the landscape of mobile phone and smartphone users in a routine public health setting. In our study in South Africa, mobile phone ownership was found to be very common among pregnant women living with HIV, with nearly all (582/637, 91.4%) surveyed owning mobile phones and 86.7% (503/580) owning smartphones. The finding of mobile phone penetration among this population is consistent with existing literature among people living with HIV and pregnant women living with HIV in South Africa [26,27]. These findings are also comparable to basic mobile phone and smartphone penetration in the general South African population [28]. With the influx of low-cost smartphone models, smartphone penetration was already over 90% in South Africa by 2019 [29]. Overall, there is a scarcity of recent literature on smartphone patterns in South Africa, particularly research that is specific to people living with HIV; our findings contribute to this knowledge. Our findings represent a sample of pregnant women living with HIV attending ANC in the public sector and are of specific interest to those developing mHealth interventions for similar populations. Also, we anticipate that smartphone ownership among pregnant adults would resemble that of nonpregnant adults, so our findings should be generalizable to all adults attending routine clinical care in this setting.

In our study, some sociodemographic characteristics differed by smartphone ownership. Consistent with a national survey and early literature, we found that smartphone owners had higher education and employment than nonowners [27,30]. We did not find significant differences in age, nor by place of birth. Researchers considering mHealth studies in similar settings should be aware that they may miss potentially vulnerable populations with lower education and employment levels by requiring smartphones.

mHealth interventions are not without their challenges, particularly in low-resource settings. Earlier research showed that some challenges of mHealth interventions in Sub-Saharan Africa include inconsistent electricity and network coverages, phone sharing, poor phone quality and battery life, as well as high mobile phone and phone number turnover [31]. A 2015-2016 mHealth study in South Africa found that 14.3% (506/3540) of screened individuals did not own a working mobile phone [32]. Furthermore, of those who owned mobile phones, a majority did not meet the technical requirements for basic app installation: 59.3% (2100/3540) of them did not use the Android operating system, 6.3% (222/3540) of them did not have the correct Android version (the app required Android version 4.2 or above), 3.8% (133/3540) of them did not have adequate storage space, and 6.4% (226/3540) of them used mobile phones that were not data enabled [32]. In our findings, however, most of the smartphones met the most basic technical requirements for installation of a mobile app. As expected, nearly all (421/458, 91.9%) of smartphones used the Android operating system, and 95.9% (485/506) of them were reported to require ≤1 battery charges per day, which is an encouraging finding for future mHealth interventions in periurban settings. Nearly all (497/506, 98.2%) smartphones included a GPS feature that is conducive for location-based mHealth interventions, such as CareConekta. Our findings indicate improvement in the quality of mobile phones and meeting of technical requirements in low-resource settings in recent years, which is encouraging for the future of mHealth. Additionally, the COVID-19 pandemic normalized many digital health applications, such as telehealth, case management, and contract tracing, which likely will accelerate the development of mHealth applications worldwide [33,34].

We also found that phone sharing was not common in this setting. Furthermore, in nearly all the cases where phone sharing occurred, the phone was being shared with a romantic partner or family member and the phone was with the owner for most of the day. These findings are consistent with the literature on phone sharing patterns in similar populations [35-37]. Limited phone sharing is encouraging for mHealth interventions because it minimizes periods of loss of engagement with the intervention, as well minimizing opportunities for privacy and confidentiality loss. This is particularly important for interventions centered around HIV management, especially given the persistence and complexities of HIV-related stigma in countries like South Africa; a common barrier to HIV adherence [38]. Although phone sharing was found to be uncommon, mHealth interventions should remain sensitive to issues of user privacy and confidentiality.

We found moderate turnover of device and phone numbers, consistent with a 2013 study on mobile phone ownership patterns among pregnant women living with HIV in South Africa [37]. However, compared to the same study, which reported that most participants had used their current phone number for 3 years and some as long as 13 years, we found slightly higher turnover [37]. Furthermore, the earlier study found that most participants used the same phone number in the past 2 years, we found that most of our participants had used 2 numbers in the past 2 years [37]. Therefore, phone and phone number turnover needs to be considered in future mHealth interventions in similar settings. This includes designing mHealth applications that are easy and cheap to reinstall, as well as easy to reregister and flexibility to access preexisting profiles.

Participants reported that making and receiving calls, as well as sending and receiving text messages, were their most used features. This is consistent with an earlier study in this setting that found SMS text messaging was the most commonly used feature [26]. Texting can be leveraged for the most basic mHealth interventions, for example, health education or adherence reminder interventions, that require minimal-to-no-direct reciprocal engagement from the individual. Furthermore, this feature is commonly found in any mobile phone, thus having the potential to reach both smartphone users and non–smartphone users.

GPS and email usage were the least common features used. Low email usage is contrary to the qualitative studies by Mogoba et al [36] and Clouse et al [37] in the same setting that showed email to be among the top smartphone uses but consistent with quantitative findings that found email usage to be the least common. Email usage or the ownership of an email address, at the very least, is important to consider for mobile app mHealth interventions because installation from platforms such as Google Play store requires a Google-authenticated email address.

We also found that the existing messaging platform WhatsApp is incredibly popular in this setting, with Facebook coming in a distant second. This finding is consistent with literature in similar settings [36,37]. Future mHealth interventions may wish to leverage the popularity of these already existing apps since people are already familiar with installing and using them and are already contributing financially to use these apps.

### Strengths and Limitations

These findings should be viewed with an understanding of the strengths and limitations of our research. The CareConekta study specifically enrolled pregnant women who were living with HIV and attending ANC; it is possible that other care-seeking individuals differ in smartphone ownership and use patterns. The CareConekta study, however, approached every pregnant women living with HIV who was attending ANC and thus this analysis is likely to be representative of this specific population of women. Missing data on eligibility checklist forms at the beginning stages of the CareConekta study resulted in the exclusion of some data (n=22) for analysis in this research. Data and airtime cost may influence smartphone ownership and usage, but we did not collect this information at enrollment and are unable to report it here. Last, a strength of this research is that we explored the technical specifications of these smartphones which is not common in the literature and provides more data to support future mHealth intervention design.

### Conclusions

In conclusion, we found that smartphone ownership is very common among pregnant women living with HIV in this low-resource, periurban setting. Common mHealth challenges such phone sharing, poor battery life to support mHealth applications, and smartphone and phone number turnover were either uncommon or moderate. These results are encouraging to the development and roll out of mHealth interventions in similar settings. Our findings on the popularity of existing platforms, particularly WhatsApp, are also encouraging and should be leveraged for mHealth interventions.

## Appendix

Multimedia Appendix 1 Smartphone usage patterns in South Africa.

## Abbreviations

ANC: antenatal care
mHealth: mobile health
MOU: Midwife Obstetric Unit

## Data Availability

Deidentified data are available upon reasonable request.

## References

[1] Petroc Taylor (2021). Forecast number of mobile users worldwide from 2020 to 2025. Statista.

[2] Forrest JI, Wiens M, Kanters S, Nsanzimana S, Lester RT, Mills EJ (2015). Mobile health applications for HIV prevention and care in Africa. Curr Opin HIV AIDS.

[3] Mechael PN (2009). The case for mHealth in developing countries. Innov Technol Gov Glob.

[4] Lester RT, Ritvo P, Mills EJ, Kariri A, Karanja S, Chung MH, Jack W, Habyarimana J, Sadatsafavi M, Najafzadeh M, Marra CA, Estambale B, Ngugi E, Ball TB, Thabane L, Gelmon LJ, Kimani J, Ackers M, Plummer FA (2010). Effects of a mobile phone short message service on antiretroviral treatment adherence in Kenya (WelTel Kenya1): a randomised trial. Lancet.

[5] Finitsis DJ, Pellowski JA, Johnson BT (2014). Text message intervention designs to promote adherence to antiretroviral therapy (ART): a meta-analysis of randomized controlled trials. PLoS One.

[6] Coleman J, Eriksen J, Black V, Thorson A, Hatcher A (2020). The mobile alliance for maternal action text message-based mHealth intervention for maternal care in South Africa: qualitative user study. JMIR Hum Factors.

[7] Hightow-Weidman L, Muessig K, Knudtson K, Srivatsa M, Lawrence E, LeGrand S, Hotten A, Hosek S (2018). A gamified smartphone app to support engagement in care and medication adherence for HIV-positive young men who have sex with men (AllyQuest): development and pilot study. JMIR Public Health Surveill.

[8] Ahmed I, Ahmad NS, Ali S, Ali S, George A, Saleem Danish H, Uppal E, Soo J, Mobasheri MH, King D, Cox B, Darzi A (2018). Medication adherence apps: review and content analysis. JMIR Mhealth Uhealth.

[9] UNAIDS (2021). Confronting inequalities: lessons for pandemic responses from 40 years of AIDS. 2021 UNAIDS Global AIDS Update.

[10] UNAIDS (2021). Country factsheets - South Africa. UNAIDS AIDSinfo 2021.

[11] Knettel BA, Cichowitz C, Ngocho JS, Knippler ET, Chumba LN, Mmbaga BT, Watt MH (2018). Retention in HIV care during pregnancy and the postpartum period in the option B+ era: systematic review and meta-analysis of studies in Africa. J Acquir Immune Defic Syndr.

[12] Clouse K, Pettifor A, Shearer K, Maskew M, Bassett J, Larson B, Van Rie A, Sanne I, Fox MP (2013). Loss to follow-up before and after delivery among women testing HIV positive during pregnancy in Johannesburg, South Africa. Trop Med Int Health.

[13] Coleman J, Bohlin KC, Thorson A, Black V, Mechael P, Mangxaba J, Eriksen J (2017). Effectiveness of an SMS-based maternal mHealth intervention to improve clinical outcomes of HIV-positive pregnant women. AIDS Care.

[14] Pop-Eleches C, Thirumurthy H, Habyarimana JP, Zivin JG, Goldstein MP, de Walque D, MacKeen L, Haberer J, Kimaiyo S, Sidle J, Ngare D, Bangsberg DR (2011). Mobile phone technologies improve adherence to antiretroviral treatment in a resource-limited setting: a randomized controlled trial of text message reminders. AIDS.

[15] Joseph-Davey D, Ponce W, Augusto O, Traca D, Jetha E, de Palha de Sousa C (2013). Improved retention in HIV care following SMS reminders in Mozambique: a randomized controlled trial. https://www.researchgate.net/publication/268389187_Improved_retention_in_HIV_care_following_SMS_reminders_in_Mozambique_a_randomized_controlled_trial.

[16] Dean AL, Makin JD, Kydd AS, Biriotti M, Forsyth BWC (2012). A pilot study using interactive SMS support groups to prevent mother-to-child HIV transmission in South Africa. J Telemed Telecare.

[17] Ippoliti NB, L'Engle K (2017). Meet us on the phone: mobile phone programs for adolescent sexual and reproductive health in low-to-middle income countries. Reprod Health.

[18] Odeny TA, Bukusi EA, Cohen CR, Yuhas K, Camlin CS, McClelland RS (2014). Texting improves testing: a randomized trial of two-way SMS to increase postpartum prevention of mother-to-child transmission retention and infant HIV testing. AIDS.

[19] Mwapasa V, Pro G, Chinkhumba J, Mukaka M, Kobayashi E, Stuart A, Gunda A, Joseph J, Sugandhi N, Chimbwandira FM, Eliya M (2014). Mother-infant pair clinic and SMS messaging as innovative strategies for improving access to and retention in eMTCT care and option B+ in Malawi: a cluster randomized control trial (the PRIME study). J Acquir Immune Defic Syndr.

[20] Schwartz SR, Clouse K, Yende N, Van Rie A, Bassett J, Ratshefola M, Pettifor A (2015). Acceptability and feasibility of a mobile phone-based case management intervention to retain mothers and infants from an option B+ program in postpartum HIV care. Matern Child Health J.

[21] Peter JE, Barron P, Pillay Y (2015). Using mobile technology to improve maternal, child and youth health and treatment of HIV patients. S Afr Med J.

[22] Kalamar AM, Bayer AM, Hindin MJ (2016). Interventions to prevent sexually transmitted infections, including HIV, among young people in low- and middle-income countries: a systematic review of the published and gray literature. J Adolesc Health.

[23] Gous N, Fischer AE, Rhagnath N, Phatsoane M, Majam M, Lalla-Edward ST (2020). Evaluation of a mobile application to support HIV self-testing in Johannesburg, South Africa. South Afr J HIV Med.

[24] Kruse C, Betancourt J, Ortiz S, Valdes Luna SM, Bamrah IK, Segovia N (2019). Barriers to the use of mobile health in improving health outcomes in developing countries: systematic review. J Med Internet Res.

[25] Myer L, Phillips T, Manuelli V, McIntyre J, Bekker L-G, Abrams EJ (2015). Evolution of antiretroviral therapy services for HIV-infected pregnant women in Cape Town, South Africa. J Acquir Immune Defic Syndr.

[26] Nachega JB, Skinner D, Jennings L, Magidson JF, Altice FL, Burke JG, Lester RT, Uthman OA, Knowlton AR, Cotton MF, Anderson JR, Theron GB (2016). Acceptability and feasibility of mHealth and community-based directly observed antiretroviral therapy to prevent mother-to-child HIV transmission in South African pregnant women under option B+: an exploratory study. Patient Prefer Adherence.

[27] Crankshaw T, Corless IB, Giddy J, Nicholas PK, Eichbaum Q, Butler LM (2010). Exploring the patterns of use and the feasibility of using cellular phones for clinic appointment reminders and adherence messages in an antiretroviral treatment clinic, Durban, South Africa. AIDS Patient Care STDS.

[28] Okano JT, Ponce J, Krönke M, Blower S (2022). Lack of ownership of mobile phones could hinder the rollout of mHealth interventions in Africa. Elife.

[29] Mzekandaba S (2020). SA’s smartphone penetration surpasses 90%. IT Web.

[30] Johnson C, Silver L (2018). Internet connectivity seen as having positive impact on life in sub-saharan Africa. Pew Research Center.

[31] Betjeman TJ, Soghoian SE, Foran MP (2013). mHealth in Sub-Saharan Africa. Int J Telemed Appl.

[32] Venter W, Coleman J, Chan VL, Shubber Z, Phatsoane M, Gorgens M, Stewart-Isherwood L, Carmona S, Fraser-Hurt N (2018). Improving linkage to HIV care through mobile phone apps: randomized controlled trial. JMIR Mhealth Uhealth.

[33] Nachega JB, Leisegang R, Kallay O, Mills EJ, Zumla A, Lester RT (2020). Mobile health technology for enhancing the COVID-19 response in Africa: a potential game changer?. Am J Trop Med Hyg.

[34] Asadzadeh A, Kalankesh LR (2021). A scope of mobile health solutions in COVID-19 pandemics. Inform Med Unlocked.

[35] Lalla-Edward ST, Mashabane N, Stewart-Isherwood L, Scott L, Fyvie K, Duncan D, Haile B, Chugh K, Zhou Y, Reimers J, Pan M, Venkatraman M, Stevens W (2022). Implementation of an mHealth app to promote engagement during HIV care and viral load suppression in Johannesburg, South Africa (iThemba Life): pilot technical feasibility and acceptability study. JMIR Form Res.

[36] Mogoba P, Phillips TK, Myer L, Ndlovu L, Were MC, Clouse K (2019). Smartphone usage and preferences among postpartum HIV-positive women in South Africa. AIDS Care.

[37] Clouse K, Schwartz SR, Van Rie A, Bassett J, Vermund SH, Pettifor AE (2015). High mobile phone ownership, but low internet and email usage among pregnant, HIV-infected women attending antenatal care in Johannesburg. J Telemed Telecare.

[38] Perez A, Brittain K, Phillips N, Stein DJ, Zar HJ, Myer L, Hoare J (2022). HIV-related stigma and psychological adjustment among perinatally HIV-infected youth in Cape Town, South Africa. AIDS Behav.

