# Supplementary material for: Smartphone Ownership and Usage Among Pregnant Women Living With HIV in South Africa: Secondary Analysis of CareConekta Trial Data
Source: JMIR Form Res. 2023 Jun 22;7:e43855. doi: 10.2196/43855 (PMC10337307; doi:10.2196/43855)
Supplement: Multimedia Appendix 1 [file formative_v7i1e43855_app1.docx]

**SMARTPHONE OWNERSHIP AND USAGE AMONGST PREGNANT WOMEN LIVING WITH HIV IN SOUTH AFRICA**

*Supplementary Appendices:*

*Appendix A: Eligibility checklist*

We are conducting a study to assess a new smartphone application (“app”) called CareConekta. We designed CareConekta to help postpartum women living with HIV link to HIV care services if they travel away from their home area. We want to assess the feasibility and acceptability of using this new smartphone app and also see if it helps to link women to care.

*Senza isifundo esibizwa ngeCareConekta sokuvavanya i-application (i-app) entsha yezifowuni zalamaxesha. Sakhe iCareConekta ukuncenda amabhinqa asanda kubeleka aphila nentsholongwane kagawulayo (i-HIV) adityaniswe neenkonzo zononophelo lwe HIV xa behambele kude kunamakhaya wabo. Sifuna ukuqonda ukufikeleleka nokwamkeleka kokusetyenziswa kwale-app intsha yeefowuni zalamaxesha, kwaye sijonge ukuba iyanceda ukudibanisa amabhinqa nononophelo lwempilo.*

If you think you might be interested, I will ask you a few demographic questions, and then check that you are eligible for the study and then we will give you all the information you need to decide if you would like to take part. Participation is voluntary and you can decide not to take part at any time without any penalty.

*Ukuba ucinga ukuba unganomdla, ndizakubuza imibuzo embalwa emalunga nawe, emva koko ndijonge ukuba ungakwazi ukuthatha inxaxheba kwesisifundo na. Emva koko sizakukunika yonke inkcazelo oyidingayo ukuze uthathe isigqibo sokuba uyafuna ukuthatha inxaxheba na. Ukuthatha inxaxheba kungokukhulu ukuzikhethela kwaye ungathatha isigqibo sokungathathi nxaxheba nangaliphi na ixesha ngaphandle kokohlwaywa.*

|  | We will be recruiting 200 women to try out this new application for about 9 months. The app will be loaded on your smartphone for free – do you think you might be interested?  Sizakumema amabhinqa ayi-200 ukuba azame le-app intsha isithuba esingangeenyanga eziyi-9. I-app izaku fakwa kwifowuni yakho yalamaxesha mahala – xa ucinga unganomdla? | | ___ Yes Ewe  ___ No Hayi → continue to demographic questions |
| --- | --- | --- | --- |
| 2. | Date screening completed  *Umhla we-screening* | | __ __ \| __ __ __ \| __ __ __ __ |
| *Eligibility screening* | | | |
| 3. | How old are you?  *Mingaphi iminyaka yakho?*  [Confirm using clinic folder or ID document]  *[Qinisekisa kwiclinic folder neID]* | | __________ years  If not yet 18 years, NOT ELIGIBLE |
| 4. | Do you own a mobile phone?  *Ingaba unayo ifowuni?* | | 0=No NOT ELIGIBLE  1=Yes CONTINUE |
| 5. | Do you own a smartphone?  *Unayo ifowuni yalamaxesha?*     - Does it have a touch screen? *Ine* *touch screen*?      - Can you use it to browse the internet? *Uyakwazi ukuyisebenzisela ujonga i-internet?*      - Is the operating system Android, version 5.0 or later? *Isebenza nge operating system ka-Android version 5.0 okanye iversion yakutsha kunoko?*   - **Android version number:**     _____________________      - Is the service provider Vodacom, Cell-C, Telkom or MTN? *Umnikezi nkonzo ngu Vodacom, Cell-C, Telkom okanye MTN?*      - Can the phone use GPS to show current location (open Google Maps or other map app). *Ifowuni iyakwazi usebenzisa i-GPS ukubonisa indawo okuyo (vula uGoogle Maps okanye enye i-app yemap)*      - Does the battery require charging less than twice a day, on average? *I-battery yefowuni idinga ukugcwaliswa ngaphantsi kwakabini ngosuku, ngokomlinganiselo? Ewe/Hayi*      - Is your phone with you today?   *Uyiphethe ifowuni yakho namhlanje?* | ___ Yes *Ewe*  ___ No *Hayi*  ___ Yes *Ewe*  ___ No *Hayi*  ___ Yes *Ewe*  ___ No *Hayi*  ___ Yes *Ewe*  ___ No *Hayi*    ___ Yes *Ewe*  ___ No *Hayi*  ___ Yes *Ewe*  ___ No *Hayi*  ___ Yes *Ewe*  ___ No *Hayi*    ___ Yes *Ewe*  ___ No *Hayi* | If ANY 0=No →NOT ELIGIBLE  If ALL 1=Yes → CONTINUE |
| 6. | Confirm by looking at her phone  *Qinisekisa ngokujonga ifowuni yakhe* | | 0=not confirmed  1=confirmed  → CONTINUE |
| 7. | Are you ≥28 weeks pregnant?  *Ingaba uneeveki eziyi-28 okanye ngaphezulu ukhulelwe?*  [Confirm in maternity case record/ *Qinisekisa kwimaternity case record]* | | 0=No → NOT ELIGIBLE – invite to review again at next clinic visit  1=Yes → CONTINUE |
| 8. | Are you HIV-positive?  *Ingaba uphila nentsholongwane kagawulayo?*  [Confirm in maternity case record] | | 0=No →  NOT ELIGIBLE  1=Yes →  CONTINUE |
| 9. | What is your preferred language?  *Ukhetha oluphi ulwimi?* | | 1=isiXhosa  2=English  3=Other, specify: _______________________ |
| 10. | Are you able to read basic isiXhosa or English?  *Uyakwazi ukufunda isiXhosa okanye isingesi sezinga elisisisekelo?*   - Confirm by asking the enrolee to read the following sentence in English or isiXhosa:   *Qinisekisa ngomcela ukuba afunde esisivakalisi silandelayo ngesiXhosa okanye ngesiNgesi:*  “I can look up a new clinic on the phone.”  “Ndingakhangela ikliniki entsha emnxebeni.” | | 0=No → NOT ELIGIBLE  1=Yes → CONTINUE |
| 11. | Based on the above questions is she eligible for study?  *Ngokwalemibuzo ingentla, angathatha inxaxheba?* | | 0=No → Continue to demographic questions  1=Yes → refer to study team for informed consent |

*Appendix B: Demographics Questionnaire*

| **Demographics of women who are ineligible or declined**  ***Iinkcukacha zamabhinqa angakwazi okanye alileyo ukuthatha inxaxheba*** | | |
| --- | --- | --- |
| Even though you are not eligible for the study or prefer not to take part, I would like to ask you a few basic demographic questions. These answers will be anonymous and will be used to describe the group of women who did not participate in the study.  Is that OK with you?  *Nokuba awukwazi ukuthatha inxaxheba okanye ukhetha ukungathathi nxaxheba, ndingathanda ukubuza imubuzo embalwa engenkcazelo yakho.*  *Ingaba uyakuvuma oku?* | | ___ Yes → Continue  ___ No →Thank you for your time! |
| ***Demographic information*** | | |
|  | How old are you?  *Mingaphi iminyaka yakho?* | __________ years |
|  | Are you currently employed?  *Ingaba uyaphangela ngoku?* | ___ Yes  ___ No |
|  | What is the highest level of schooling that you have completed?  *Leliphi elona nqanaba liphezulu lokufunda/lemfundo olugqibileyo?* | ___ Never completed primary school (did not finish grade 7/standard 5  *Zange ndiwagqibe amabanga aphantsi (zange ndimgqibe ugrade 7/ustandard 5)*  ___Completed primary school only (finished grade 7/standard 5)  *Ndiwagqibe amabanga aphantsi (Ndimgqibile ugrade 7/ustandard 5)*  ___Some high school *Amanye amabanga aphezulu* (grade 8-11/standard 6-9)  ___Completed high school *Ndiwagqibile amabanga aphezulu* (matric/grade 12/standard 10)  ___Any tertiary education *Nayiphi na imfundo ephakamileyo* |
|  | Where were you born?  *Wazalelwa phi?* | ___In Western Cape (*eNtshona Koloni)*  ___In South Africa, but not Western Cape (*eMzantsi Afrika, kodwa hayi eNtshona Koloni)*  *Specify province:*____________________________  Not in South Africa (Hayi eMzantsi Afrika)  *Specify country:* ____________________________ |
|  | Write phone make/model and IMEI number  *Bhala uhlobo lwefouni nenombolo ye IMEI* | Make:___________________________  Model:__________________________  IMEI:____________________________ |
|  | Why would you not like to participate? [only for those who did not wish to participate]  *Kutheni ungenothanda ukuthatha inxaxheba?* |  |
